# Lung Adenocarcinoma Transcriptomic Analysis Predicts Adenylate Kinase Signatures Contributing to Tumor Progression and Negative Patient Prognosis

**DOI:** 10.3390/metabo11120859

**Published:** 2021-12-09

**Authors:** Jonathan A. Chacon-Barahona, Ivan A. Salladay-Perez, Nathan James Lanning

**Affiliations:** 1 Department of Biological Sciences, California State University, Los Angeles, CA 90032, USA; jchaco16@calstatela.edu (J.A.C.-B.); isallad@calstatela.edu (I.A.S.-P.); 2 Molecular Biology Interdepartmental Program, University of California, Los Angeles, CA 94701, USA

**Keywords:** adenylate kinase, HIF-1, hypoxia, lung cancer, tumor progression

## Abstract

The ability to detect and respond to hypoxia within a developing tumor appears to be a common feature amongst most cancers. This hypoxic response has many molecular drivers, but none as widely studied as Hypoxia-Inducible Factor 1 (HIF-1). Recent evidence suggests that HIF-1 biology within lung adenocarcinoma (LUAD) may be associated with expression levels of adenylate kinases (AKs). Using LUAD patient transcriptome data, we sought to characterize AK gene signatures related to lung cancer hallmarks, such as hypoxia and metabolic reprogramming, to identify conserved biological themes across LUAD tumor progression. Transcriptomic analysis revealed perturbation of HIF-1 targets to correlate with altered expression of most AKs, with AK4 having the strongest correlation. Enrichment analysis of LUAD tumor AK4 gene signatures predicts signatures involved in pyrimidine, and by extension, nucleotide metabolism across all LUAD tumor stages. To further discriminate potential drivers of LUAD tumor progression within AK4 gene signatures, partial least squares discriminant analysis was used at LUAD stage-stage interfaces, identifying candidate genes that may promote LUAD tumor growth or regression. Collectively, these results characterize regulatory gene networks associated with the expression of all nine human AKs that may contribute to underlying metabolic perturbations within LUAD and reveal potential mechanistic insight into the complementary role of AK4 in LUAD tumor development.

## 1. Introduction

Amongst the most common cellular characteristics of lung cancer, hypoxia within the tumor microenvironment plays a pervasive set of roles that affect primary tumor development through altered bioenergetic metabolism [1,2,3]. To date, the most widely studied molecular driver of cellular hypoxic response is hypoxia-inducible factor-1 (HIF-1), a highly conserved transcription factor that exerts oxygen mediated control on glucose and oxidative metabolism through modulating global transcriptome expression [4,5,6,7]. Clinically, it is difficult to diagnose tumor hypoxia. However, the incorporation of transcriptome analysis has provided a new model for diagnosis. HIF-1 expression, and its gene targets, have shown to be molecular biomarkers for tumor hypoxia [8,9]. Therefore, it is important to understand both the progression of HIF-1 signaling throughout cancer and associations with HIF-1 and bioenergetic sensitive enzymes. Adenylate kinases (AKs) represent a family of bioenergetic sensitive enzymes that are emerging contributors to cancer etiology and progression [10,11]. Nine human AK isoforms (AK1–9) are known to date. The AKs are key enzymes that buffer adenine nucleotide ratios [2ADP ←→ATP + AMP]. Perturbed expression of AKs has been shown to modulate global energy-sensitive signaling pathways under hypoxic conditions in lung cancer cells and macrophages [12,13,14]. Collectively, understanding the global interactions between HIF-1 signaling and AK expression in lung cancer can provide new methods to detect the pathology and progression of the disease and provide new insights into studying the mechanistic interaction between HIF-1 and AK.

Structurally, HIF-1 exists as a heterodimer composed of an oxygen-sensitive HIF-1α and nuclear-translocating HIF-1ß subunits [15,16]. Under normoxic conditions, cytoplasmic HIF-1α undergoes constant ubiquitination in response to hydroxylation of P402 and P577 residues by the HIF prolyl hydroxylases (PHDs) [17,18,19]. Thus, hydroxylated HIF-1α is recurringly targeted for proteasomal degradation [20,21,22]. By contrast, cytoplasmic HIF-1ß, under normoxic conditions, remains constitutively expressed and poised for interaction with HIF-1α [23,24]. Upon depletion of intracellular oxygen levels, HIF-1α ubiquitination and subsequent degradation steps are inhibited, thus facilitating HIF-1α binding to HIF-1ß which is followed by translocation of the resulting HIF-1 transcription factor to the nucleus [25,26]. Here, HIF-1 binds transcription-enhancing promoter regions of over 100 genes, largely through recognition of hypoxia response elements scattered throughout the genome [5,27,28]. This pathway collectively describes a canonical mechanism of HIF-1 activation and emphasizes the importance of HIF-1α stability in HIF-1 downstream signaling.

Alternatively, elevated reactive oxygen species (ROS) have been shown to inhibit HIF-1α destabilization, even under normoxic conditions [29]. This is thought to occur through ROS-mediated inhibition of PHDs, which interrupts oxygen-PHD interactions, effectively restricting PHD-induced hydroxylation of HIF-1α [30]. Additional HIF-1-inducing pathways have been described in a nonhypoxic setting, which involves cell supplementation with hormones and growth factors [31], or deficits in SIRT3 and JunD transcription factors [32,33]. Notwithstanding, these nonhypoxic avenues for HIF-1 signaling collectively depend on elevated ROS levels.

Modulation of AK isoform levels has also been demonstrated to impact ROS levels within the context of cancer metabolism. For example, deficits in AK2 levels are associated with increased ROS production and decreased glycolysis and ATP production [34]. Furthermore, overexpression of AK3 has been shown to augment ROS production in squamous cell carcinoma cells treated with cisplatin [35]. By contrast, within the context of colorectal cancer, AK6 has been shown to promote decreased cellular ROS via the Warburg effect [36].

Recently, the mitochondrial-localized adenylate kinase 4 (AK4) has been shown to augment intracellular ROS production to promote HIF-1α stability, and by extension HIF-1 signaling, in the context of macrophages, breast cancer, and lung adenocarcinoma (LUAD) [13,14,37]. Additionally, given that AK4 has previously been described as a relatively novel target of active HIF-1 [38], the relationship between AK4 and HIF-1 adheres to that of a positive feedback loop, where modulation of AK4 levels impacts cellular metabolism, ROS production, and HIF-1 regulation. Moreover, similar to HIF-1, AK4 has been shown to promote chemotherapeutic resistance in tumors and is regarded as an unfavorable prognostic marker for tumor metastasis and lung cancer patient outcomes [37,39,40,41,42].

In this study, we use LUAD patient primary tumor transcriptome data across stages 1–4 to analyze AK co-expression signatures that reveal potential AK4-driven hallmarks of tumor development and metastasis. Co-expression analysis of all AK isoforms reveals AK4 transcript levels to increasingly correlate with a LUAD-specific hypoxia signature throughout early LUAD tumor development, leading up to metastasis. Furthermore, principal component analysis (PCA) of LUAD patient AK expression patterns reveal AK4 to carry a distinctive gene signature that—when grouped with the nearest clustering AKs—serves as an unfavorable LUAD patient prognostic marker. Finally, in order to identify conserved biological themes related to AK4 transcript expression in LUAD, a comprehensive LUAD tumor AK4 co-expression network was constructed of stage-specific tumor AK4 gene signatures, encompassing both early (stage 1/2) and late (stage 3/4) developmental milestones of LUAD tumor growth and metastasis. This interrogation across LUAD stages provides a richer analysis that is also more faithful to accepted LUAD stages compared to previous publications. Enrichment analysis of these signatures implicates increasingly perturbed nucleotide metabolism throughout LUAD tumor development. Moreover, the use of sparse partial least squares discriminant analysis (sPLS-DA) against LUAD tumor stage-to-stage interfaces within the AK4 co-expression network identified candidate genes that may contribute to LUAD tumor growth or regression. Additionally, LUAD patient survivorship analysis of these candidate genes largely validated the sPLS-DA results and revealed that even favorable anti-oncogenic prognostic markers could be associated with increased patient mortality when accounting for AK4 expression. Collectively, these results expand the scope of influence that AK4 exerts on pathological LUAD tumor dynamics.

## 2. Results

### 2.1. Baseline Patient Sample Characteristics

This study assessed 526 patients with LUAD. Classically, with a data set this large, there can be an overrepresentation of a unique variable over another. Therefore, to obtain a better understanding of patient characteristics, an R-based function was used to tally the number of patient samples that belong to various patient characteristics (Table 1). The patient characteristics used for this study were age, gender, vitals, tumor stage, sample type, and race. Moreover, 295 patients were over or equal to the age of 60, 128 patients were under 60 years of age, and 103 were not reported. For gender, there is a small difference in the number of females (*n* = 282), over males (*n* = 244). For vitals, there is a substantial difference in the number of patients that are alive at the time of biopsy (*n* = 336), compared to dead (*n* = 190). For tumor stage, a concomitant decrease in samples that represent progressed tumors was observed. Of the 526 patients, 59 submitted biopsies from peritumoral tissues, which were pooled together to form a control group Lastly, there was a dramatic difference among races with white representation making up the majority of the TCGA-LUAD cohort.

### 2.2. Evidence of Sustained Hypoxic Response through HIF-1 Signaling in LUAD

**Figure 1:**
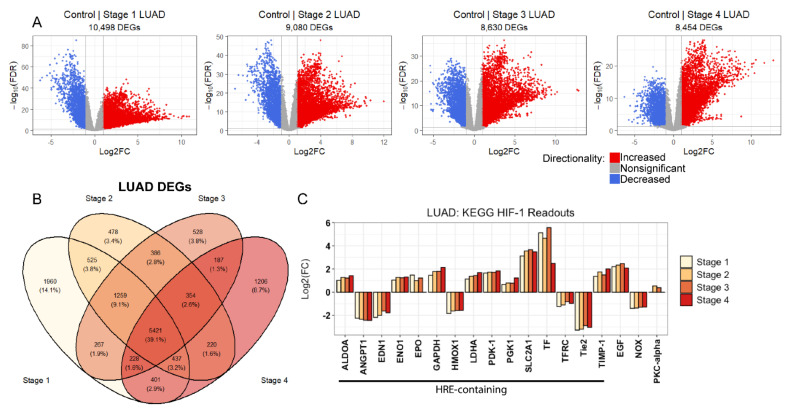
Evidence of a sustained hypoxic response through HIF-1 signaling in LUAD. (**A**) Volcano plot showing differential transcript expression across LUAD tumor stage 1–4. A significance threshold of ≥ 2-fold change (FDR < 0.05) reveals immense differential transcript expression at LUAD tumor stage 1–4 transcriptomes relative to non-tumorous controls. (**B**) Venn diagram depicting conserved and stage-specific differentially expressed genes across LUAD tumor development. (**C**) LUAD tumor stage 1–4 differentially expressed genes for angiogenesis and metabolism-modulating genes within the canonical KEGG HIF-1 signaling readouts reveals continuous and extensive perturbation of downstream Hypoxia Response Element (HRE)-containing transcripts.

Using a significance threshold of ≥ 2-fold change (FDR < 0.05), we found remarkable differential transcript expression across the LUAD tumor stage 1–4 transcriptomes relative to non-tumorous human lung tissue controls (Figure 1A, Supplementary Table S1). Furthermore, while ~40% of all detected differentially expressed genes were identified at all LUAD tumor stages, there were large sets of unique differentially expressed genes found exclusively at specific tumor stages or stage-stage intersections (Figure 1B). Nonetheless, we found conserved and extensive perturbations across the KEGG HIF-1 signaling pathway for HIF-1 signaling readouts at all LUAD tumor stages (Figure 1C, Supplementary Table S2). These readouts approximate upstream and downstream effectors of HIF-1 signaling, with downstream effectors, in particular, containing hypoxia response elements in their promoter—directly recruiting HIF-1 transcriptional regulation. Thus, perturbations in these readouts are consistent with elevated HIF-1 signaling through the transcriptional upregulation of well-known HIF-1-responsive genes involved in promoting increased anaerobic metabolism, such as glyceraldehyde 3-phosphate dehydrogenase (GAPDH), solute carrier family 2 member 1 (SLC2A1), hexokinase (HK), lactate dehydrogenase A (LDHA), phosphoglycerate kinase 1 (PGK1), aldolase A (ALDOA), and enolase 1 (ENO1) [43,44,45,46,47,48,49,50,51,52,53]. Likewise, we found significant transcriptional upregulation for readouts of augmented HIF-1 signaling that promotes increased oxygen delivery through erythropoiesis and angiogenesis, including erythropoietin (EPO), epidermal growth factor (EGF), and tissue inhibitor matrix metalloproteinase 1 (TIMP-1) [54,55,56], along with increased transcript expression of pyruvate dehydrogenase kinase 1 (PDK-1)—a known inhibitor of tricarboxylic acid (TCA) cycle metabolism [57].

With regard to down-regulated genes involved in angiogenesis within the canonical HIF-1 signaling pathway, angiopoietin 1 (ANGPT1)—a putatively favorable serum prognostic marker for non-small cell lung cancer [58]—had suppressed expression across LUAD tumor at stages 1–4. Similarly, we observed down-regulated transcript levels in the ANGPT1 receptor Tie2, which itself is regarded as a favorable prognostic marker for liver and renal cancers (Human Protein Atlas). Given the combined roles of ANGPT1 and Tie2 in negatively regulating angiogenesis and vascular permeability, suppressed expression of these transcripts, in the context of upregulated EGF and TIMP-1 transcripts, predicts pro-angiogenic signaling along the HIF-1 signaling axis that runs throughout the early and late stages of LUAD tumor development. Additionally, the HIF-1-regulated vascular tone-modulating endothelin 1 (EDN1) and heme oxygenase 1 (HMOX1) transcripts were significantly suppressed throughout the entirety of LUAD tumor development. Importantly, these transcript expression changes were largely consistent throughout LUAD tumor development, alongside significantly suppressed levels of intermittent hypoxia-mediating NADPH oxidase (NOX) transcripts. It is also important to note that this suppression coincided with modest, but insignificant, increases in the transcript levels of protein kinase C-α (PKC-α), another readout of intermittent hypoxia [59,60], only at LUAD tumor stages 2 (1.44-fold increase; FDR = 0.003) and 3 (1.310-fold increase; FDR = 0.025). Thus, these transcriptional perturbations describe canonical HIF-1 signaling that resembles an intratumoral state of sustained hypoxia in response to acute or chronic, as opposed to intermittent, oxygen deficiency.

### 2.3. AK Levels Positively Correlate with Hypoxia Scores throughout LUAD Tumor Progression

**Figure 2:**
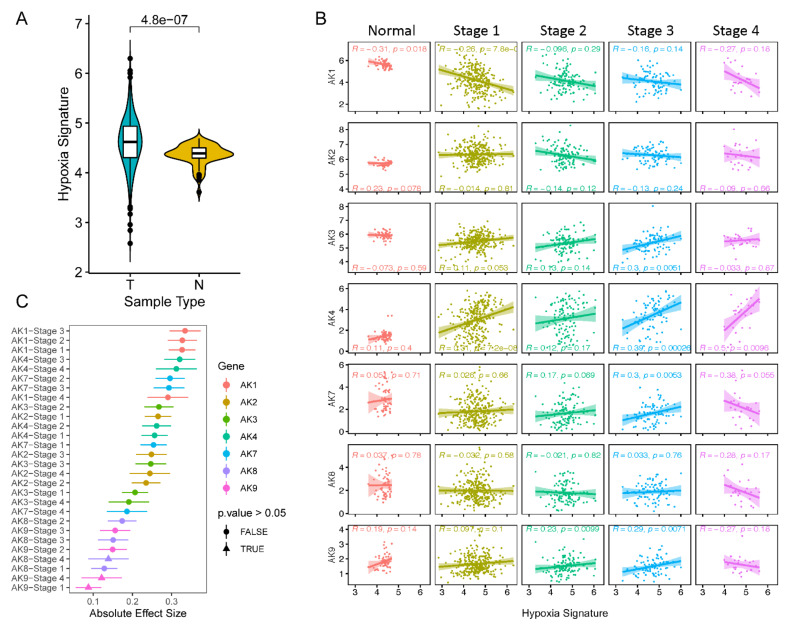
Intertumoral correlations between a hypoxia-associated gene signature and AK gene expression. (**A**) Gene signature is made up of the median expression of four genes XPNPEP1, ANGPTL4, SLC2A1, and PFKP. Tumor samples are represented in blue and normal samples are in yellow (*t*-test, *p* = 4.8 × 10^7^). (**B**) AK correlation with the hypoxia score among tumor stages. Colors represent the different groups in cancer (red = Normal, yellow = Stage 1, green = Stage 2, blue = Stage 3, pink = Stage 4). R = Pearson correlation coefficient and *p*-values represent significant interactions between hypoxia and AK expression. (**C**) Effect of tumor-specific interaction compared to peritumoral normal tissue type. Linear regression modeling was used to quantify the significant absolute effect size between normal and tumor specific interactions.

To determine the relationship between AK expression and hypoxia in LUAD, an established hypoxia-associated gene signature was curated and normalized to tissue-specific differences. The genes include: *XPNPEP1*, *ANGPTL4*, *SLC2A1*, and *PFKP*. This selection was obtained from a previous study by Mo et al., where they identified these genes as good predictors of hypoxia, poor patient outcomes, and tumor size [47]. Tissue type-specific gene expression distributions were mined and normalized using the same methods described above for AK isoforms and the hypoxia signature (Figure S1 and Figure 2A). We tested for differences in expression levels between the normal (N) and the tumor type (T). It was observed that seven of the nine AKs have significant differences compared to the normal control group (Figure S1). The median expression of the four genes is classified as a “Hypoxia Signature.” Similar to the study by Mo et al., the tumor hypoxia signature has more variation compared to the normal and its median expression value is significantly increased (Figure 2A). This signature was then used as a dependent variable to assess the relationship between HIF-1α and AK expression. Linear-based regression modeling was used to compare the significance between the two at each LUAD tumor stage, including the peritumoral normal tissue type (Figure 2B). For this section, AK5/6 was omitted due to its non-normal distribution. Using Spearman’s correlation coefficients, we found the hypoxia signature to correlate with seven of the nine AK isoforms across multiple stages of LUAD tumor development. The highest coefficient found among these interactions was an AK4-stage 4 specific interaction (R = 0.50, *p* = 0.0096) which suggests a biologically relevant relationship between AK4 and hypoxia. Lastly, a pair-wise comparison, derived from two-way linear regression modeling, between normal and stage-specific interactions was assessed to calculate the gene-stage specific absolute effect size between stages and its normal tissue type control (Figure 2C). AK8/9 showed no significant differences in effect between normal and stage four tumor interactions with hypoxia. This was also seen for AK9 only in a stage one interaction. This data provides key insights into a potential mechanistic axis in which AK isoforms are reprogrammed in LUAD.

### 2.4. Two AK Clusters Predict Poor LUAD Patient Prognosis

**Figure 3:**
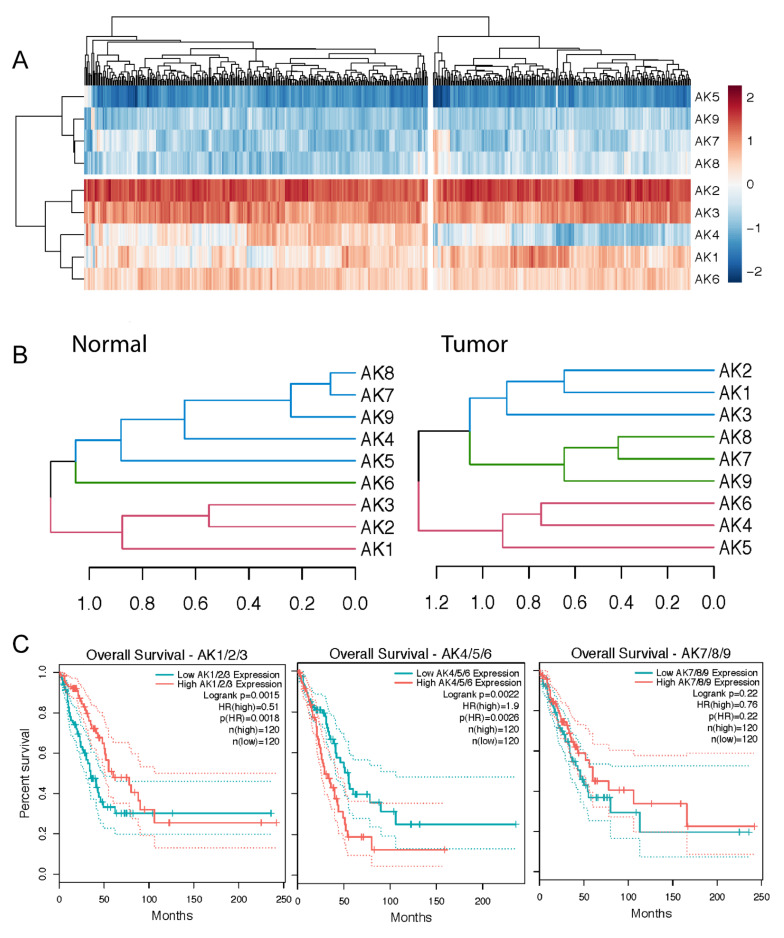
Differential gene expression of adenylate kinases form clusters that predict poor patient outcomes. (**A**) Heatmap representing gene expression data from tumor samples. Gene expression values represent a z-score from −2 to +2 using a complete linkage method with Euclidean distance. Dendrograms were split into two top clusters across genes (rows) and patients (columns). (**B**) AK hierarchical clusters. Dendrograms represent the relationship between genes within normal (**left**) and tumor (**right**) data using complete linkage method using dissimilarity measures. Colors highlight three ranked gene clusters within the normal and tumor data and x-axis represents dissimilarity between gene expression. (**C**) Survival analysis based on normalized gene expression of the three AK clusters using a log-rank test and 95% confidence intervals. 75% and 25% quartile ranges were used to split the patient groups into high and low expression groups. HR represents hazard ratio and “n” represents the number of patients used for the analysis.

Seven of the nine AK isoforms correlated with a previously described hypoxia-associated gene signature. Therefore, it is necessary to elucidate AK gene expression patterns within LUAD and distinguish key differences across tissue types (normal vs tumor). To understand the relationship of AKs in LUAD, we scaled Log2 (TPM + 1) values into a z-score, clustered genes along the y-axis, and clustered 526 patient tumor samples along the x-axis. We identified two types of AK groups in LUAD: overexpressed (AK1–4/6) and low-expressed (AK5/7–9) (Figure 3A). It was also observed that AK4 has a unique expression pattern that split tumor samples into two major clusters. This moved us to assess whether there were any significant changes in AK4 expression, along with the other AKs, within tumor stages. To do this, patients were grouped by tumor stage and an ANOVA analysis determined significant differences between stages for each AK isoform (Figure S2A). Three of the nine isoforms demonstrated significant changes between tumor stages (ANOVA, p* <0.05), with AK4/7 expression appearing to increase and AK9 expression to decrease with tumor stage. To further understand correlation patterns between these and other AKs, Pearson’s correlation coefficients were calculated to assess the similarity of each pair of genes in normal and tumor tissue profiles (Figure S2B). Hierarchical clustering using Euclidean distances of each gene further classified which genes cluster together among the normal and tumor tissues (Figure 3B). The top three clusters are distinguished by color and the results demonstrate a shift in AK expression patterns moving from the normal to tumor sample types. To determine whether there is a clinical relevance to these clusters, Kaplan–Meier estimates were used to measure the fraction of patients who survive LUAD as a function of the median expression of each gene cluster. The upper 75th percentile of gene expression was used for the high expressed group and the lower 25th percentile was used to assess low expression. Interestingly, two of the three clusters had a significant interaction with patient survivability of LUAD (Figure 3C). Normalized z-score quantification (Figure 3A) suggests AK4 to have unique expression patterns across LUAD compared to other AKs. Dendrograms demonstrate a shift in expression from the normal to tumor tissues and the AK1–3 and AK4–6 cluster was shown to significantly predict poor survivability outcomes. Together, this data highlights the direction in AK expression patterns and provides new insights into what AK expression looks like in LUAD with a unique highlight on AK4 expression.

### 2.5. AK4 Expression Network Comprised of Perturbed Nucleotide Metabolism in LUAD

**Figure 4:**
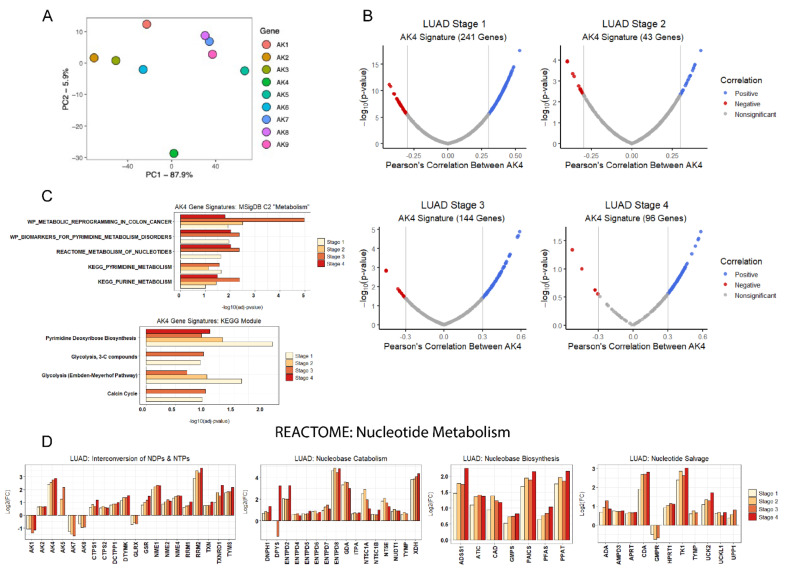
Stage-specific AK4 gene signatures implicate perturbed regulation of nucleotide metabolism in LUAD. (**A**) Bulk expression-based unsupervised clustering via PCA identifies AK4 to have unique expression patterns compared to other AKs in LUAD. (**B**) LUAD tumor stage-specific AK4 gene signatures were created by identifying genes that co-express with AK4 using a Pearson’s correlation coefficient threshold of ± 0.3. (**C**) Over-representation analyses of these AK4 gene signatures reveals that AK4 co-expresses with genes involved in nucleotide metabolism at all stages of tumor development. (**D**) LUAD stage 1–4 differential transcript expression of genes involved in the four REACTOME nucleotide metabolism subgroups (FDR < 0.05).

Given the previously reported biological significance of AK4 in lung cancer [13,14], along with unique isoform-specific expression patterns revealed in Figure 2 and Figure 3, we sought to use an unsupervised clustering algorithm to parse AK expression in tumor tissue types (Figure 4A). It was observed that the AK4/5/6 group, unlike the others, does not cluster when AK transcript expression is projected to describe AK-specific variance via PCA. In fact, AK4 expression appeared to be distant from other AK isoforms suggesting a unique expression pattern. Therefore, we sought to characterize AK4-related co-expression networks across LUAD tumor stages 1–4. Here, we adopt a modified approach towards characterizing LUAD stage-specific AK4 gene signatures based on Jan et al.’s use of Pearson’s correlation coefficients [13], with the key differences being that our approach encompasses the entirety of LUAD tumor progression using TPM-normalized transcript estimates and additionally excludes genes with no stage-specific significant difference in transcript expression relative to control non-tumorous lung tissue. Using this approach, we constructed AK4 gene signatures across LUAD stage 1–4 differentially expressed genes, effectively creating a LUAD tumor AK4 co-expression network (Figure 4B). Interestingly, this AK4 co-expression network contained many hypoxia response element-containing HIF-1 signaling readouts scattered throughout different LUAD tumor stages, including: ALDOA (r > 0.4), ANGPT1 (r < −0.3), ENO1 (r > 0.4), GAPDH (r > 0.3), LDHA (r > 0.3), PDK1 (r > 0.4), SLC2A1 (r > 0.3), and TRFC (r > 0.3) (Supplementary Table S3). Nonetheless, to characterize individual gene signatures, we additionally used each LUAD stage-specific signature as inputs for over-representation analysis (ORA) within two curated databases: KEGG Modules, and the Broad Institute’s Molecular Signature Database C2 collection—both of which contain diverse gene sets related to metabolic processes. ORA of these gene signatures suggests the AK4 co-expression network comprises genes related to nucleotide metabolism, like previous reports (Figure 4C). Indeed, further characterization of the perturbed expression of transcripts involved in nucleotide metabolism across LUAD stages 1–4 overwhelmingly reveals significant upregulation of transcripts involved in multiple facets of nucleotide metabolism, most of which further deviate from that of control tissue levels as LUAD tumor development continues (Figure 4D).

### 2.6. AK4 Co-Expression Networks Identify Potential Drivers of LUAD Tumor Progression

**Figure 5:**
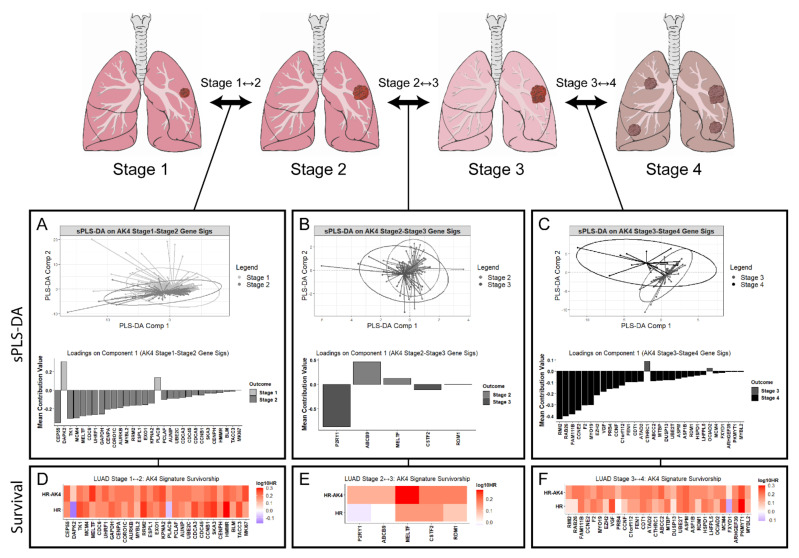
Sparse partial least squares-discriminant analysis (sPLS-DA) of AK4 gene signatures at LUAD tumor stage-stage interfaces allows for discrimination of potential drivers underlying LUAD tumor progression within the AK4 co-expression network. differentially expressed genes within AK4 gene signatures contributing to a specific tumor stage outcome are predicted at LUAD tumor (**A**) stage 1–2, (**B**) stage 2–3, and (**C**) stage 3–4 interfaces. GEPIA2 LUAD patient survival analysis was performed using single genes (represented with “HR”) and pairwise AK4-containing gene signatures (represented with “HR-AK4”) as inputs to validate the sPLS-DA results at the (**D**) stage 1–2, (**E**) stage 2–3, and (**F**) stage 3–4 LUAD tumor interfaces. Log_10_-transformed hazard ratios (HRs) are shown.

Using these AK4 co-expression networks, we next sought to identify potential drivers of LUAD tumor progression using a staggered classification approach to predict genes that contribute to tumor growth or regression at tumor stage-to-stage interfaces (Figure 5). Here, we opted to employ sPLS-DA using LUAD tumor stage-specific AK4 gene signatures to extract key genes capable of discriminating tumor progression or regression. sPLS-DA of merged LUAD tumor stage-to-stage AK4 signatures generated a list of gene candidates with ranked contributions to tumor advancement or regression. Across stage 1–2, 2–3, and 3–4 interfaces, the majority of key LUAD tumor stage discriminating genes were associated with cell cycle regulation and mitosis, DNA processing and repair, and chromatin remodeling processes.

Unsurprisingly, the putative tumor suppressor gene death associated protein kinase 2 (DAPK2) was predicted to greatly contribute to tumor regression at the LUAD tumor stage 1–2 interface (Figure 5A). Interestingly, at the stage 1–2 AK4 gene signature interface, the pyrimidine salvage protein thymidine kinase 1 (TK1) was predicted to be amongst the most influential for LUAD tumor progression from stage 1 to stage 2. Furthermore, the P2Y purinergic receptor 1 (P2RY1), which has been shown to serve as an unfavorable prognostic marker in renal and non-melanoma skin cancers ([61], (Human Protein Atlas)), is also predicted to promote LUAD tumor progression at the stage 2–3 interface (Figure 5B). Additionally, the use of AK4 LUAD tumor gene signatures is associated with the oncogenic protein RAD52 motif Containing 1 (RDM1) at the LUAD tumor stage 2–3 and 3–4 interfaces (Figure 5C). This aligns with RDM1′s previously described role as a pro-oncogenic factor [62]. Notably, at the LUAD tumor stage 3–4 interface, RDM1 was predicted to contribute to LUAD tumor advancement from stage 3 to stage 4.

To independently validate the sPLS-DA results, we used the GEPIA2 web tool (http://gepia2.cancer-pku.cn/#index (accessed on 9 November 2020)) to perform a LUAD patient survivorship analysis using single genes (e.g., DAPK1, TK1, etc.) and pairwise AK4-containing gene signatures (e.g., AK4-DAPK2, AK4-TK1, etc.) as inputs (Figure 5D–F). Overall, sPLS-DA predictions aligned well with the inferred survivability of each tested gene, as indicated by Log_10_-transformed hazards ratios. In particular, survivorship analysis of early LUAD tumor regression candidates at the stage 1–2 interface (e.g., DAPK2 and PLAC9) revealed these genes to serve as positive LUAD prognosis markers (Figure 5A,D). Additionally, at all LUAD tumor stage-to-stage interfaces, there was an overwhelming amount of consensus between genes in the AK4 gene signatures that—via sPLS-DA—predicted LUAD tumor advancement and the survival analysis.

When pairwise AK4-containing gene signatures were used as inputs for LUAD patient survival analysis, all AK4 combinations resulted in poor patient prognosis (Figure 5D–F). Interestingly, of the few genes that, via sPLS-DA, were predicted to contribute to tumor regression at certain stage-to-stage interfaces—including DAPK2, PLAC9, ABCB9, MELTF, CTHRC1, and OCIAD2, testing for LUAD patient survivability via AK4 combination resulted in a negative prognosis. This was especially true for DAPK2 and PLAC9, which individually associated with improved patient prognosis, but reverted to a poor patient prognostic marker when combined with AK4 for LUAD patient survival analysis.

## 3. Discussion

While it has long been known that limited intratumoral oxygen availability impacts tumor metastatic potential, in part through the transcription regulatory actions of HIF-1, both spatial and temporal intratumoral hypoxia dynamics impact tumor development, chemotherapeutic resistance, and cell seeding; providing dramatically different cancer patient outcomes [63]. In particular, the relationship between chronic or acute hypoxia and lung cancer tumor metastatic potential has specifically been investigated, revealing that acute hypoxia and concomitantly high HIF-1α stability most strongly increase tumor growth and metastasis [64]. Here, we report perturbed transcriptional regulation of hypoxia response element-containing genes within the KEGG HIF-1 signaling pathway that is largely conserved throughout LUAD tumor pathogenesis and consistent with chronic or acute, as opposed to intermittent, hypoxia (Figure 1C, Supplementary Table S2). This is evidenced by two observations: the first being that differential transcript expression of HIF-1 signaling readouts was observed as early as LUAD tumor stage 1 and persisted throughout the entirety of LUAD tumor development. In particular, we observed HIF-1 pathway perturbations to fit the profile of a chronic hypoxia response and also reflect known hallmarks of HIF-1 signaling in lung cancer, including increased anaerobic metabolism and angiogenesis, and suppressed TCA cycle metabolism. The second observation relates to the significant suppression of NOX at all LUAD tumor stages (Figure 1C). In the context of intermittent hypoxia, NOX has been shown to mediate HIF-1α activation, and HIF-1 has been shown to promote the expression of NOX [65,66]. Thus, the observed profile of HIF-1 hypoxia response element-containing readouts and significantly suppressed NOX expression suggests that intermittent hypoxia is not occurring within LUAD patient tumors and that, instead, chronic or acute hypoxia is featured throughout the early and late stages of LUAD tumor pathology.

The median expression levels of four target genes of HIF-1 were calculated as our hypoxia score. Individually, the expression of these genes is altered under anaerobic stress conditions in vitro. Here, like many studies, we show that there is elevated transcription of hypoxia-associated genes in solid tumors, such as in lung adenocarcinomas [67]. We used this model as a marker for tumor progression at a metabolic response level. For example, tumors with a higher propensity for growth and cell seeding often carry increased levels of a global hypoxic signature [63,64]. The expression of AK transcripts was revealed to be significantly different between normal and bulked LUAD tumor tissues and their expression in cancer correlates with this hypoxic score. Notably, an AK1/4 co-expressed signature was previously characterized in LUAD patients [12]. That study demonstrated an opposite correlation between AK1 expression and survival and AK4 expression and survival (high AK1 expression is correlated with increased survival while low AK4 expression is correlated with increased survival). In our present study, we confirm and extend these results by demonstrating two findings: (1) within the entire AK family, there are three clusters of AKs whose LUAD expression patterns closely associate (Figure 3B); (2) these clusters may be clinically relevant as their combined expression patterns (low versus high expression within each cluster) are associated with significantly different survival probabilities (Figure 3C). Our results also recapitulate the Jan et al. study in that the low expression of the AK1 cluster is correlated with increased survival while low expression of the AK4 cluster is correlated with increased survival. We (Lanning et al.) and others (Hao et al.) have also previously published that low AK4 expression correlates with increased survival in gliomas and pancreatic cancer. We also report comprehensive AK signatures that parse out the associations between individual AK isoform expression and a hypoxia score throughout control lung and LUAD tumor stage 1–4 tissues (Figure 2). In doing so, we reveal a dynamic association between AK transcript expression and hypoxia in the context of LUAD tumor development, specifically highlighting an increasingly positive correlation between AK4 levels and hypoxia as LUAD tumor development continues. This finding is consistent for stages 1, 3, and 4, but is variable for stage 2 (Figure 2B). This finding, which aligns with previous LUAD-related AK4 research [13,68], also describes a progressively negative prognostic role for AK4 in LUAD. Interestingly, unsupervised clustering identified AK4 to have unique expression patterns independent of other AKs, including AK1, which highlighted the reason to further characterize AK4 in LUAD. AK4 was also shown to interact with HIF-1 signaling under hypoxic conditions in m1 macrophages which further supports the interaction to be a global response in a tumor microenvironment [14].

While disrupted glucose metabolism has long served as a hallmark of many cancers, including lung cancer [69,70], a systems-level focus on perturbed nucleotide metabolism in cancer and the underlying associated mechanisms has only recently obtained broader attention. By adopting an AK4-centric approach towards understanding the link between AK4 and its widely reported role as a negative prognostic marker, we report the AK4 co-expression network to potentially explain a facet of perturbed nucleotide metabolism in LUAD related to purine and pyrimidine synthesis, catabolism, and salvage (Figure 4B–D). Using our prior knowledge of LUAD patient tumor stage, we additionally incorporated this network—comprised of individual AK4 gene signatures—to predict genes that contribute to tumor progression or regression via sPLS-DA (Figure 5A–C). Here, validation of the sPLS-DA predictions was completed using single gene LUAD patient survivorship analysis, and by relying on prognostic information available in the literature. As a whole, the predictions made by sPLS-DA aligned well with the associated survivability for each tested gene, especially at early LUAD stage-stage interfaces, such as the LUAD tumor stage 1–2 interface, which includes DAPK2 and PLAC9 (Figure 5A,D). The reason for this may be that sPLS-DA identifies key transcript determinants that drive discrimination of tumor stage interfaces, whereas survival analysis looks for overall survivability. Therefore, regardless of whether a gene is predicted to promote tumor regression at later LUAD tumor stage interfaces, this predicted regression still points in the direction of a later LUAD tumor stage, and thus, is marked as an unfavorable prognostic marker via survival analysis. Nonetheless, we found it interesting that when testing for LUAD patient survivability using an AK4 combination, all resulting survivorship hazard ratios were associated with increasing mortality (Figure 5D–F). This finding was especially intriguing when considering that the associated survivability of positive prognostic markers, such as DAPK2 and PLAC9, was essentially reversed when testing for LUAD patient survivability using an AK4-DAPK2 and AK4-PLAC9 signature. Thus, the use of AK4 expression in gene signatures for LUAD patient survivability may reveal more about the deleterious impact that AK4 has on the dynamic LUAD pathology.

Additionally, the use of AK4 gene signatures with sPLS-DA identified the nucleotide salvage gene TK1—another negative prognostic marker of lung cancer (Figure 5A)—to co-express with AK4, and promote LUAD tumor progression as previously reported [71,72]. Interestingly, both AK4 and TK1 have been described to function within a larger nucleotide-protein interaction network [73]. When looking at LUAD patient survivability, high expression of TK1 and a TK1-AK4 signature are also associated with increased patient mortality (Figure 5D). Given that AK4 has been shown to worsen lung cancer patient outcomes by suppressing levels of activating transctiption factor 3 (ATF3) [40], a transcriptional regulator of TK1 [74], our finding that TK1 promotes LUAD tumor progression within the larger AK4 co-expression network may expand on the mechanistic link between AK4, ATF3, and TK1 in the context of LUAD. In particular, suppression of ATF3 levels by AK4 may inhibit ATF3 regulation of TK1, permitting up-regulation of TK1 in LUAD. Figure 5C also identified RDM1 association with the AK4-driven signature across tumor stages. Thus, our AK4-driven signature identifies known pro-oncogenic factors, lending credibility to our signature, and also suggests previously unidentified tumor stage associations with these known pro-oncogenic factors.

Importantly, these findings are limited to the site of LUAD patient tumor biopsy retrieval, and the concomitant variation that arises when accounting for intratumoral heterogeneity [75,76]. Similarly, it is worth noting that the LUAD tumor transcriptomes available for use in this study were overwhelmingly from Caucasian participants, which surmised a majority of the available LUAD TCGA cohort. Nonetheless, this report expands on the associations between perturbed AK isoform expression and LUAD hypoxic status, and collectively reveals potential mechanistic insight into how AK4 serves as a negative prognostic marker in LUAD tumor development.

## 4. Materials and Methods

Unless otherwise specified, all analyses were performed using RStudio v1.3.959, © 2009–2020

### 4.1. TCGA Patient Tumor Data Curation and Consolidation

TCGA data query was performed using TCGAbiolinks v2.17.4 and MultiAssayExperiment v1.14–0 packages to obtain patient tumor-matched RNA-seq and counts data through the NIH funded Genome Data Commons domain [77,78]. The biomaRt v2.44.4 package was used to match ensemble gene ID to gene symbols [79].

### 4.2. Gene Expression Query, Normalization, and Quantification

In this study, gene expression quantification data in fragments per kilobase of transcript per million mapped reads (FPKM) were queried using a TCGA harmonized database which aligns reads to the human reference genome 38 (hg38). All FPKM data were standardized to transcripts per million (TPM) to adjust for the measurement of gene expression as a proportion of transcripts in the total pool of RNA. The equation is recited below (1). Once data were standardized to TPM, a $\log_{2}\left(TPM+1\right)$ transformation was used to normalize the data into a normal distribution for appropriate statistical analysis.

(1) $$TPM_{i}=\frac{FPKM_{i}}{\sum_{j}FPKM_{j}}\times 10^{6}$$

### 4.3. Statistical Analysis

All statistical analyses, apart from the survival plots, were conducted locally in RStudio. All pairwise *t*-test comparisons, two-way ANOVAs, and Pearson’s correlation analysis were computed using the ggpubr package. Statistically different groups scored a *p*-value less than 0.05. Linear regression and unsupervised clustering analysis were completed with base R functions: lm( ) and prcomp( ). Linear modeling tested the interaction between hypoxia signature and AK expression. AK5/6 were excluded from linear modeling for their non-normal distributions. Principal component analysis (PCA) was performed using prcomp( ). Principal components were calculated separately for each AK in tumor RNA-seq profiles. PC1 and PC2 explained 93.8% of the variation and were therefore selected for visualization. Survival plots were generated from a web-based expression interactive tool known as Gene Expression Profiling Interactive Analysis (http://gepia.cancer-pku.cn/). High and low gene expression groups were split using the top 75th and lower 25th percentiles. Visualization of hierarchical clustering output was performed using ggdendro v0.1.22.

### 4.4. Hypoxia Signature

The median expression of four genes, *XPNPEP1, ANGPTL4, SLC2A1,* and *PFKP*, was used in this study to estimate the hypoxic status of tumor samples. The four genes make up a hypoxia-associated gene signature and were selected amongst 200 other hypoxia-associated genes to predict patient outcomes, tumor hypoxia, and pathological stage in lung adenocarcinoma samples as previously described [47].

### 4.5. Differential Gene Expression Analysis

TCGA participant-derived HTSeq counts were assembled into LUAD stage-specific and control collections using the cloud-based Galaxy environment (https://usegalaxy.org). Within this environment, differential gene expression for a specific LUAD stage relative to control was estimated using edgeR v3.24.1 [80]. Given the prior uncertainty in gene dispersion within the numerous tumor and control samples, we opted to use the quasi-likelihood F-test edgeR parameter to determine differential expression [81]. We additionally filtered out lowly expressed genes with less than 10 total counts and applied a *p*-value adjustment to obtain the false discovery rate (FDR) using the Benjamini and Hochberg method [82]. For curated gene ID mapping, we used the Bioconductor clusterProfiler package v3.18.1 to map Ensembl IDs against Entrez IDs and excluded non-mapped values from further analysis [83]. Entrez-mapped differential transcript expression was considered significant if transcripts had ≥ 2-fold difference relative to control at an FDR < 0.05.

### 4.6. Co-expression Network Creation and Over-Representation Analysis

AK4 gene signatures were created using an adapted form of the gene co-expression construction guide presented by Contreras-López et al., whereby variance in TPM counts is standardized and summed up a unit value to avoid zeroes [84]. Furthermore, this approach included only significant differentially expressed genes for each LUAD stage group using the significance threshold described above. Finally, stage-specific AK4 gene signatures were created by taking the Pearson’s correlation coefficient between AK4 and all other differentially expressed genes within a specific LUAD stage group and applying a threshold of ± 0.3. Collectively, we found this approach—motivated by the approach previously accomplished by Jan et al. [13], to discriminate AK4 co-expression networks by LUAD stage and additionally incorporate later progression of LUAD tumor development with the inclusion of stage 3–4 tumor samples.

Using genes within the stage-specific AK4 co-expression networks as inputs, we performed ORA against gene sets within KEGG Modules and the Broad Institute’s Molecular Signature Database C2 Collection [85,86], each of which containing curated gene sets that are functionally related to biological process or state. All ORA were also performed using clusterProfiler v3.18.1.

### 4.7. Sparse Partial Least Squares-Discriminant Analysis

To identify candidate genes within the LUAD stage-specific AK4 co-expression networks, we use an extended sparse version of partial least squares regression analysis termed sPLS-DA within the mixOmics R package v6.14.0 [87,88,89]. Here, two sequential LUAD stage AK4 co-expression networks were incorporated in each sPLS-DA analysis to determine the mean contribution value of AK4 gene signature components to the LUAD tumor stage. All sPLS-DA parameter inputs were estimated using the mixOmics’ parameter tuning functions. The optimal number of variables (i.e., genes) per component was determined using a data-driven one-sided *t*-test approach that evaluates changes in model performance as additional variables are incorporated into the model. This approach was validated under maximum distance via M-fold cross-validation at 50 repeats for each sPLS-DA analysis.

## Figures and Tables

**Table 1:** Patient demographics.

Variable	TCGA-LUAD Cohort (*n* = 526 Patients)
**Age**	
<60	128
≥60	295
Not reported	103
**Sex**	
Female	282
Male	244
**Vitals**	
Deceased	190
Alive	336
**Tumor Stage**	
1	316
2	135
3	97
4	28
Not reported	9
**Sample Type**	
Solid tissue normal (N)	59
Primary tumor (T)	526
**Race**	
American Indian or Alaska Native	1
Asian	7
Black or African American	54
White	398
Not reported	66

## Data Availability

No data were created during the present study. The results here are in whole or part based upon data generated by the TCGA Research Network: https://www.cancer.gov/tcga (accessed on 9 November 2020).

## Supplementary Materials

The following are available online at https://www.mdpi.com/article/10.3390/metabo11120859/s1, Figure S1: Differential Gene Expression of Adenylate Kinases. T-test comparisons between normal (N) and tumor (T) data sets, Figure S2: Expression Heterogeneity of Adenylate Kinase Among form tissue specific hierarchical Clusters, Table S1: All significant differentially expressed genes (fold change ≥ 2; FDR < 0.05) in stage 1–4 lung adenocarcinoma patient primary tumor transcriptomes, Table S2: Differential transcript expression of HIF-1 signaling readouts in lung adenocarcinoma tumor stages 1–4, Table S3: HIF-1 signaling readouts also found in stage-specific AK4 gene signatures across lung adenocarcinoma stages 1–4.
