# Supplementary material for: Lung Adenocarcinoma Transcriptomic Analysis Predicts Adenylate Kinase Signatures Contributing to Tumor Progression and Negative Patient Prognosis
Source: Metabolites. 2021 Dec 9;11(12):859. doi: 10.3390/metabo11120859 (PMC8705281; doi:10.3390/metabo11120859)
Supplement: Supplementary file 1 [file metabolites-11-00859-s001.zip › Supplementary figures.pdf]

metabolites

Article

Lung Adenocarcinoma Transcriptomic Analysis Predicts Adenylate Kinase Signatures Contributing to Tumor Progression and Negative Patient Prognosis

Jonathan A. Chacon-Barahona [1,†], Ivan A. Salladay-Perez [1,2,†] and Nathan James Lanning [1,*]

[1] Department of Biological Sciences, California State University, Los Angeles, CA 90032, USA; jchaco16@calstatela.edu (J.A.C.-B.); isallad@calstatela.edu (I.A.S.-P.)
[2] Molecular Biology Interdepartmental Program, University of California, Los Angeles, CA 94701, USA
* Correspondence: nlannin@calstatela.edu; Tel: +1-(323)-343-2092
† Authors contributed equally to this work.

SUPPLEMENTARY FIGURES

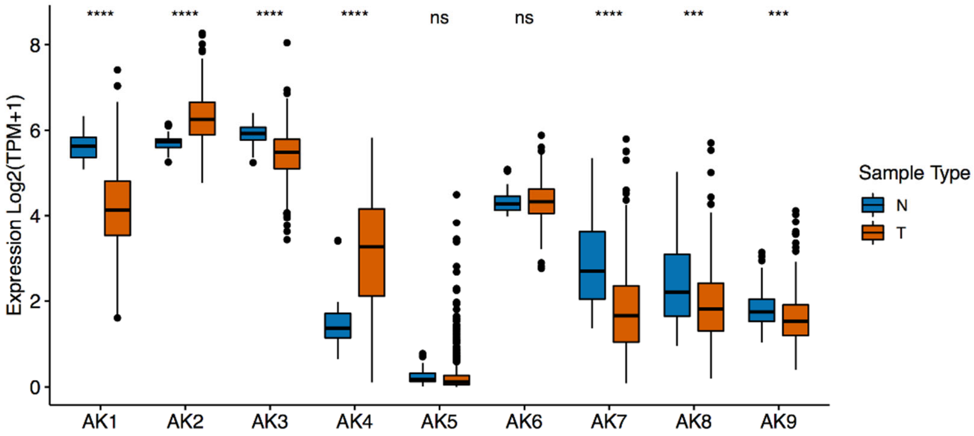

Figure S1. Differential Gene Expression of Adenylate Kinases. T-test comparisons between normal (N) and tumor (T) data sets. Multiple (⋆) indicates a P-value < 0.05.

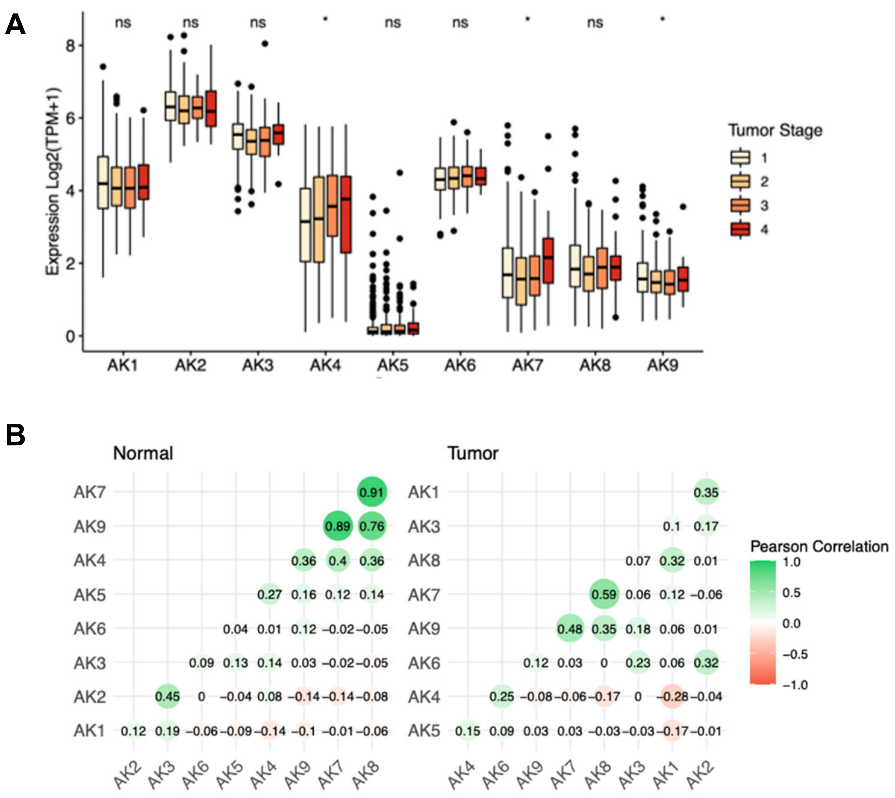

Figure S2. Expression Heterogeneity of Adenylate Kinase Among form tissue specific hierarchical Clusters. (A) RNA data was mined and modeled to look for specific gene expression changes among tumor stages (stages 1-4). ANOVA test comparisons among tumor stages. Single (⋆) indicates a P-value less than 0.05. Adenylate Kinase Hierarchical Clusters. (B) Correlation matrix for normal (left) and tumor (right) data. Correlation coefficients represent and genes were grouped based of a hierarchical clustering order.
